# Determinants of precancerous cervical lesions among women screened for cervical cancer at Debre Markos town public health facilities, Ethiopia

**DOI:** 10.1186/s12978-026-02399-6

**Published:** 2026-06-24

**Authors:** Yehualashet Belayneh, Aysheshim Asnake Abneh, Asmamaw Mengist, Tadele Derbew Kassie, Yilkal Tafere

**Affiliations:** 1 Department of Public Health, Debre Markos Comprehensive Specialized Hospital, Debre Markos, Ethiopia; 2 Department of Public Health, College of Medicine and Health Science, Debre Markos University, Debre Markos, Ethiopia

**Keywords:** Precancerous cervical lesions, Cervical cancer screening, Cervical cancer, Determinants, Ethiopia

## Abstract

**Background:**

The potential for precancerous cervical lesions to develop into cervical cancer over time is a significant public health concern in low and middle-income countries, including Ethiopia. The Ethiopian government is supporting the prevention of cervical cancer by acetic acid screening and vaccinations. However, the determinants of precancerous cervical lesions were not well studied. Therefore, this study aimed to identify determinants of pre-cancerous cervical lesions among women screened for cervical cancer at Debre Markos town public health institution, Ethiopia, 2025.

**Methods:**

An institution-based unmatched case-control study was conducted among 340 women (85 cases and 255 controls) who underwent cervical cancer screening. Cases were selected consecutively, and controls were selected using systematic random sampling. Data were collected through a structured interviewer-administered questionnaire and chart review. Data were entered using Epi-Data Version 4.6 and then transferred to STATA 15 for analysis. Descriptive statistical analysis computed frequencies and presented them with texts and tables. To determine the factors linked to precancerous cervical lesions, this study used bivariate logistic regression analysis (*P* < 0.25), multivariable logistic regression (*P* < 0.05), and odds ratios with 95% Confidence Interval (CIs). The Hosmer-Lemeshow goodness-of-fit test was used to evaluate the model’s fitness.

**Result:**

A total of 85 cases and 255 controls were included in this study, resulting in a 100% response rate. Age above 40 years adjusted odds ratio (AOR): 2.99 (1.03–8.90), rural residence AOR = 3.69 (1.46–9.36), Live alone AOR = 4.86 (2.05–11.5), history of STIs AOR = 5.3 (2.12–13.3), genital mutilation AOR = 5.98 (2.37–15.08), early initiated sexual intercourse AOR = 3.47 (1.27–9.5), having HIV positive AOR = 7.04 (2.75–17.9), and having two or more lifetime sexual partners AOR = 6.87 (2.74–17.2) were determinants of precancerous cervical lesion.

**Conclusions:**

This study highlights the importance of behavioural, biological, and socio-cultural factors in the development of precancerous cervical lesions among women in Ethiopia. HIV infection, history of sexually transmitted infections, multiple sexual partners, and female genital mutilation were identified as key determinants requiring targeted prevention and screening interventions. Strengthening integrated cervical cancer screening services may improve early detection and reduce the burden of cervical cancer in Ethiopia.

## Background

A precancerous cervical lesion (PCL) is an abnormality in the cells of the cervix that could eventually develop into cervical cancer [1]. Cervical cancer(CC) is a disease defined by an abnormal growth of cells in the cervical lining, generally starting from the squamocolumnar junction [2]. The burden of CC remains much higher than the threshold set by the World Health Organization (WHO ) initiative on CC elimination [3]. Globally, CC is a major public health issue, which ranks as the fourth leading cause of death in women [2]. In 2020, there were an estimated 604,127 CC cases and 341,831 deaths [3]. A systematic review and meta-analysis study conducted in Latin American countries showed that CC incidence ranges between 136.0 and 398.4 per 100,000 [4].

In Sub-Saharan Africa (SSA), CC is the second most frequent malignancy, where 90% of CC-related global deaths occur in this region [5]. SSA is one of the most rigorously affected regions by CC, with 117,316 new incidences and 76,745 deaths annually in 2020 [6]. Evidence showed that CC-related deaths were 18 times higher in low-and middle-income countries than in high-income countries [7].

Ethiopia is one of the SSA countries affected by CC. In Ethiopia, CC ranks the second most common cause of cancer-related deaths among women, secondary to breast cancer, requiring immediate action for screening and vaccination [1]. Nowadays, the number of CC patients diagnosed and requiring treatment in Ethiopia is significantly rising, with an estimated 29.4 million women at risk of acquiring CC [2]. Evidence suggested that the prevalence of PCL among Ethiopian women was 9.43% [8], with the East Gojjam zone having a higher prevalence than the overall estimate [9].

Human papillomavirus (HPV) is the most prevalent human viral infection, particularly in Africa, including Ethiopia, which causes 90 to 97% of CC cases [10]. PCL is caused by a variety of factors, including sociodemographic, socioeconomic, sexual and reproductive health, and medical or surgical comorbidities [11–18]. Moreover, the main factors that affect PCL are age at first sexual contact, multiple sexual partners, and HIV positivity. The weakened immune response among HIV infected women facilitates persistent HPV infection, accelerates the progression of cervical epithelial abnormalities, and increases the odds of developing PCL [19]. Furthermore, the common factors reported by the studies are older age, a history of having more than four children, and long-term use of oral contraceptives, a significant risk factor for PCL [20]. Existing evidence suggests that women who have assisted vaginal delivery, female genital mutilation (FGM), or episiotomy are at an increased risk of CC [21]. FGM is a significant risk factor for CC, particularly in African women infected with human papillomavirus [22]. The global prevalence of FGM is estimated at 36.9%, with substantially higher burden observed in SSA (56.4%). Ethiopia bears a particularly high burden, a reported prevalence of 77.3% [22–24].

The consequences of advanced stages of CC can be challenging to cure, leading to high treatment costs on the health system and communities (1) [25], reducing productivity, increasing premature death, and disability [26]. Moreover, CC survivors exhibit sexual dysfunction and a lower quality of life compared to healthy women without [27].

Although CC is preventable and treatable through vaccination, screening, and early diagnosis and treatment [3], and the Ethiopian government, in collaboration with the WHO, is implementing a CC prevention program using Visual inspection with Acetic Acid (VIA) screening for all sexually active women [28], the burden related to CC is still high in the country. Despite PCL continuing to be a significant public health concern in Ethiopia, including the study setting [29], determining factors associated with PCL, particularly FGM, episiotomy, and assisted vaginal delivery, were not well studied. Furthermore, most previous studies were cross-sectional and lacked strong evidence in identifying determinant factors between cases (women with PCL) and controls (women without PCL). Compared with cross-sectional studies, a case-control study in the current analysis provides a stronger methodological approach for investigating determinants of PCL because it enables comparison between women cases) and controls regarding their previous exposure history [30]. Identifying determinants of PCL is crucial for healthcare providers, policymakers, and program directors in developing more targeted screening programs, which could help reduce the high morbidity and mortality of CC in the country [12]. Therefore, this study aimed to identify the determinants of PCL among women screening for cervical cancer at Debre Markos town public health facilities.

### Objective

To identify independent predictors of PCL among women screened for CC at Debre Markos town public health facilities, Northwest Ethiopia, 2025, using multivariable logistic regression analysis.

## Methods

### Study design

An institution-based, unmatched case-control study design.

#### Study setting and period

This study was conducted at Debre Markos Town Public Health Institutions (Debre Markos Comprehensive Specialized Hospital, Debre Markos Health Centre, Hidase Health Centre, Waseta Health Centre, and Gozamen Health Centre). These health facilities are located 300 km from Addis Ababa and provide preventive, curative, and rehabilitative services to an estimated 3.5 million people. This study was conducted from March 15, 2025, to May 15, 2025.

### Source population

All women receiving screening for cervical cancer at the Debre Markos Town Public Health institution.

#### Study population

##### Cases 

All women having positive VIA test results in the Debre Markos Town Public Health institution during the study period.

##### Controls

All women having negative VIA test results in the Debre Markos Town Public Health institution during the study period.

### Eligibility of the study

#### Inclusion criteria

Women who had positive VIA findings for case and women who had negative VIA findings for controls were included in this study.

#### Exclusion criteria

women who were critically ill and unable to respond during the data collection period were excluded from the study.

### Sample size determination

Double population proportion sample size calculation formulas were used to estimate the minimum adequate sample size using Epi Info version 7.2.6.0 with the assumption of 95% CI, power = 80%, with a case and control ratio of 1:3, which was taken from the previous study done at Woliso town, Southwest Shoa, Ethiopia, 2022 [31]. The largest sample size from Table 1 was 307. After adding a 10% non-response rate, the final minimum adequate sample size was 340, with 85 cases and 255 controls. The details were given in Table 1.

**Table 1:** Sample size determination using EPI-Info version 7.2.6.0 at Debre-Markose town public health facility, Northwest Ethiopia, 2025

Variable	AOR	%of Cases	%of Controls	Required sample of		Total sample size	Reference
				Case	Control		
Age at first sexual intercourse	4.67	33.1	69.8	22	65	87	[18]
STI	3.02	28.6	11.7	62	185	247	[32]
Multiple sexual partners	3.75	62.3	30.58	29	86	115	[15]
Parity	2.18	61.6	42.4	77	230	307	[18]

*AOR *Adjusted Odds Ratio, *STI *Sexually Transmitted Infections

### Sampling technique and procedure

**Fig. 1:**
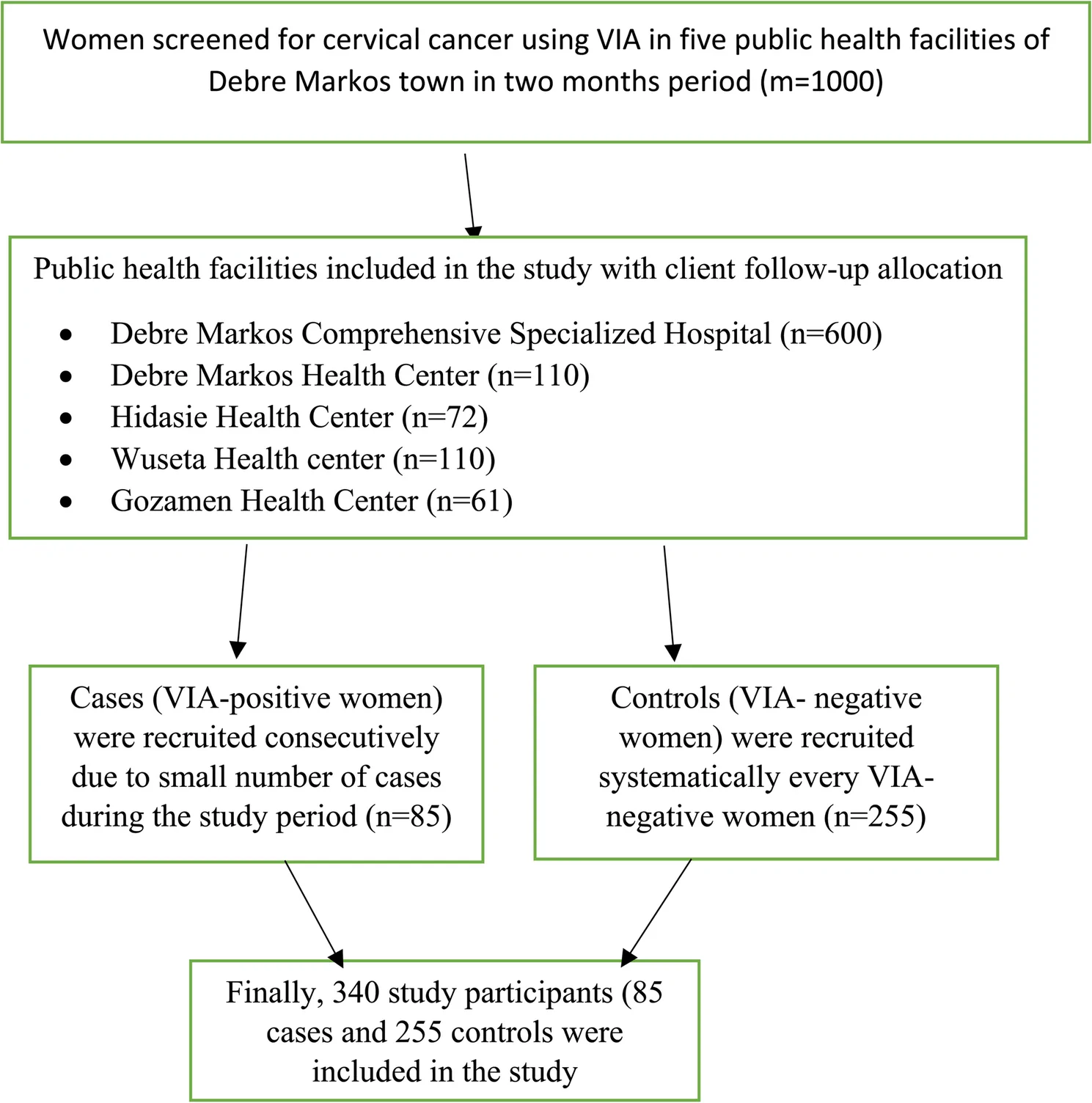
Participant flow diagram adapted for the unmatched case–control study among women screened for CC using VIA in public health facilities of Debre Markos town, Northwest Ethiopia, 2025

There are five public health facilities in Debre Markos town, and the expected average number of CC screenings per two months was 1000. The total sample sizes were allocated using probability proportionate to size, based on the proportion of average monthly client flow reported in the report. Since VIA-positive women were relatively uncommon during the data collection period (March 15 to May 15, 2025), consequent recruitment was considered the most feasible approach to fulfil the required sample size. Controls were selected systematically using a calculated K interval from women screened on the same day and at the same facilities to maintain comparability between groups. For each case, three controls (negative visual inspection with acetic acid screens) were selected during the data collection period. However, selecting controls from the same day could introduce some temporal similarity between cases and controls, and this was acknowledged in the limitation section. The average client follows, and the required sample size allocation of cases and controls in each health facility was summarized as follows: in Debre Markos Comprehensive Specialized Hospital, 204 (51 cases and 153 controls) were selected from 600 client follow-ups per two-month period. In Debre Markos Health Centre, 11 cases & 33 controls were selected from 110 clients followed. In Wuseta Health Centre, 10 cases & 30 controls were selected from the 110-client flow. In Hidase Health Centre, 7 cases and 21 controls were selected from 72 client flow, and in Gozamen Health Centre, 6 cases & 18 controls were selected from 61 client flow. Finally, a total of 85 women with VIA positive and 255 women with VIA negative were /included in the study (Fig. 1).

### Variables of the study

#### Dependent variable

Precancerous cervical lesion.

#### Independent variables

##### Socio-demographic related factor

Age, residence, marital Status, educational status, occupation, average monthly income

##### Reproductive related factor

Use of contraception, age of first birth, number of children, family history of CC, FGM, episiotomy, assisted vaginal delivery.

##### Lifestyle and sexually behavioural-related factor

History of STI, early initiation of sexual intercourse, lifetime sexual partner, HIV/AIDS result.

##### Hygienic related factor

Sharing of underwear, genital hygiene practice.

#### Operational definition

##### Precancerous cervical lesion

VIA result indicated a dense, acetowhite near the squamocolumnar junction within one minute of applying 3–5% acetic acid.

##### Case

A participant who had a positive VIA finding [33].

##### Control

A participant who had a negative VIA finding [33].

##### Multiple sexual partners

A woman who had two or more sexual partners in her lifetime [31].

##### Early age of sexual intercourse

Initiation of sexual intercourse below the age of 18 years [31].

##### Sharing of underwear

Women who shared their pants with family, friends, and other relatives [31].

##### Genital hygiene practice

In this study, genital hygiene practice refers to the self-reported practice of vaginal washing per day. Accordingly, a woman is considered to have genital hygiene practice if she reports washing of the vagina at least once per day [31].

##### Sexually Transmitted Infections (STIs)

In this study, a woman was considered to have an STI if she reported having at least one physician-diagnosed or self-reported symptom suggestive of STI during the last 12 months, including abnormal vaginal discharge, burning pain during urination, lower abdominal pain, genital itching or genital ulcer, based on WHO syndromic management guidelines [34].

##### Use of contraception

Defined as any history of using contraceptive methods, including oral contraceptives, injectables, implants, or IUCDs before or during the study.

##### Assisted vaginal delivery

Defined as vaginal delivery facilitated by instruments such as forceps or vacuum extraction.

##### Female Genital Mutilation (FGM)

Defined as the reported history of any form of female genital cutting or mutilation performed for non-medical reasons.

##### Episiotomy

Defined as a surgical cut made to the perineum during childbirth, as reported by the participant.

: 

### Data collection tools and procedures

The data were collected through a pre-tested, interviewer-administered questionnaire and maternal chart review. The data collection tool was adopted from previous literature [16, 18, 31] and had four parts, including the socio-demographics variable, reproductive-related factor, lifestyle and sexual behavioural-related factor, and hygienic-related factor. The inclusion of variables in this study was based on previous empirical evidence, biological plausibility, and prior literature established risk factors for the cervical cancer pathway. Brief instruction was given, and confidentiality was assured. To reduce the potential for recall and social desirability biases, trained female data collectors were recruited to conduct the data collection process in a private room after confidentiality was assured. Five employed BSC midwives were recruited at random to collect the data with two MSC midwife supervisors. Routine cervical cancer screening is provided to all women by trained midwives. According to the WHO guidelines for screening and treatment of precancerous lesions for cervical cancer prevention, the result can be interpreted as: positive, when an acetowhitish lesion is found; and negative when there is no acetowhitish lesion.

### Data quality assurance

To ensure data quality, the questionnaire was prepared in English and translated into Amharic and back to English by language experts to maintain its consistency. Besides, one-day training and clear orientation about the tools of this study were given to data collectors and supervisors. Furthermore, before the actual data collection, a pretest was done by taking 5% (20 participants: 5 cases, &15 controls) of the study population at Lumamie Primary Hospital, and necessary modifications were made. Reliability was assessed using Cronbach’s alpha at a p-value of 0.86. Finally, the collected data were checked for completeness, accuracy, and consistency by the principal investigator before data entry.

### Statistical analysis

After data collection, each questionnaire was checked for completeness, then coded and entered into EpiData (version 4.6) and exported to Stata version 15, software for analysis. The final data file was compiled for analysis. Descriptive statistical analysis was computed. Binary logistic regression was used to identify significant factors of a positive VIA test. A P-value of 0.25 was used as a cut-off point to screen variables considered for multivariable logistic regression. This threshold helps avoid excluding potentially important variables before multivariable analysis. The variables that were significant at a 0.05 p-value at the multivariable logistic regression were considered statistically significant associations with PCL. The model fitness was checked using the Hosmer-Lemeshow goodness of fit test (p-value 0.545), and the assumption of chi-square distribution suggested that the model adequately fit the data. Multicollinearity was checked using the variance inflation factor (IVF) at a maximum value of 8, which indicated no significant multicollinearity among independent variables. An odds ratio with a 95% confidence interval was also used to measure the degree of association between independent and dependent variables. Results were displayed in text and tables.

### Ethical consideration

The ethical approval letter was obtained from the Institute Review Board of the Debre Markos University Research Committee (protocol number: RCSTTD/455/01/17) on the date of 11/03/2025. All research procedures were conducted in accordance with the ethical standards outlined in the Declaration of Helsinki and relevant institutional and national regulations. The confidentiality of information was maintained by excluding personal identifiers and private interviews. Data were collected after securing informed consent from every respondent.

## Results

### Socio-demographic characteristics of the respondent

As presented in Table 1, this study involved a total of 340 cervical cancer-screened women, among whom 85 had PCL (cases), and 255 were controls with a 100% response rate. Among the study participants, 49 (57.6%) of cases and 116 (45.4%) of controls were found to be in the age group of above 40 years. Related to education status, 32 (37.6%) cases and 123 (48.24%) controls were unable to read and write. Related to marital status, 18 (21.18%) of cases and 169 (66.27%) of controls were married. Among study participants, 40 (47.06%) of cases and 130 (50.98%) of controls were housewives (Table 2).

**Table 2:** Socio-demographic characteristics of precancerous cervical lesions at Debre Markos public health facilities, Northwest Ethiopia, 2025 (*n* = 340)

Variables	Category	Cases, *N* (%)	Controls, *N* (%)
Age in years	≤ 30	15 (17.7)	75 (29.4)
	31–40	21 (24.7)	64 (25.1)
	> 40	49 (57.6)	116 (45.5)
Residence	Rural	64 (75.3)	96 (37.7)
	Urban	21 (24.7)	159 (62.4)
Educational status	Unable to read and write	32 (37.7)	123 (48.2)
	Primary	32 (37.7)	61 (23.9)
	Secondary	13 (15.3)	40 (15.7)
	College and above	8 (9.4)	31 (12.2)
Marital status	Live alone (single, divorced, widowed)	67 (78.8)	86 (33.6)
	Married	18 (21.2)	169 (66.3)
Occupational status	Housewife	40 (47.1)	130 (51.0)
	Merchant	9 (10.6)	35 (13.7)
	Daily laborer	16 (18.8)	37 (14.5)
	Government employee	13 (15.3)	36 (14.1)
	Private /NGO	7 (8.2)	17 (6.7)
Average monthly income	≤ 3000 Ethiopian birr	11 (12.9)	21 (8.2)
	3001–5000 Ethiopian birr	32 (37.7)	76 (29.8)
	*≥* 5001 Ethiopian birr	42 (49.4)	158 (62.0)

### Reproductive health-related characteristics of the respondent

The extent of contraceptive use was 55 (64.7%**)** and 87 (34.1%**)** among cases and controls, respectively. Regarding menstrual history, 5 (5.9%) and 11 (4.3%) of cases and controls had a history of post-coital bleeding, respectively. In addition, 63(74.1%) of cases and 174 (68.2%) of controls had ever given birth. Among study participants, 6 (7.1%) and 11 (4.3%) of controls had a family history of cervical cancer. Furthermore, 73 (85.9%) cases and 86 (33.7%) controls had genital mutilation (Table 3).

**Table 3:** Reproductive health-related factor characteristics of precancerous cervical lesions in Debre Markos public health facilities, Northwest Ethiopia, 2025 (*n* = 340)

Variables	Category	Cases, *N* (%)	Controls, *N* (%)
Ever use of a contraceptive	Yes	55 (64.7)	87 (34.1)
	No	30 (35.3)	168 (65.9)
Type of contraceptive (*n* = 55)	Injectable	20 (36.4)	38 ((43.8)
	Implant	15 (27.3)	28 (32.2)
	IUCD	9 (16.4)	6 (6.9)
	Oral	11 (20.0%)	15 (17.2)
Age at menarche	Less than 15 years old	31 (36.5)	83 (32.6)
	Greater than or equal to 15	54(63.5)	172 (67.5)
Menstrual history	Regular	16 (18.8)	107 (41.9)
	Sometimes irregular	28 (32.9)	64 (25.1)
	Always irregular	30 (35.3)	69 (27.1)
	No menses	11 (12.9)	15 (5.9)
Post-coital bleeding	Yes	5 (5.9)	11 (4.3)
	No	80 (94.1)	244 (95.7)
Ever give birth	Yes	63 (74.1)	174 (68.2)
	No	22 (25.9)	81 (31.8)
Mode of last child delivery (*n* = 237)	SVD	42 (79.3)	115 (81)
	CS	9 (14.3)	33 (18.9)
	Instrumental delivery	11 (20.8)	27 (19.1)
Parity	≤ 3	28 (44.4)	83 (47.7)
	> 3	35 (55.6)	91 (52.3)
Ae at first birth	Less than 18	10 (18.9)	39 (22.4)
	18–25	39 (61.9)	73 (41.9)
	Greater than 25	14 (22.2)	62 (35.6)
History of genital trauma at birth	Yes	27 (51.9)	62 (53.0)
	No	25 (48.0)	55 (47.0)
History of abortion	Yes	20 (23.5)	73 (28.6)
	No	65 (76.5)	182 (71.4)
Number of abortions (*n* = 93)	1–2	18 (21.2)	69 (27.1)
	3 and above	2 (2.4)	4 (1.6)
Family history of CC	Yes	6 (7.06)	11 (4.3)
	No	79 (92.9)	244 (95.7)
FGM	Yes	72 (85.9)	86 (33.7)
	No	12 (14.1)	169 (66.3)

The mode of last child delivery was calculated among 237 women who had ever given birth

### Sexual and behavioural characteristics of study participants

Among study participants, of 73(85.88) cases, 139(54.51) of the controls had first sexual intercourse Less than 18 years. Regarding sexually transmitted infections (STIs), 69(81.18) of cases and 101(39.69) of controls had a history of STI. In terms of HIV status, 40(47.06) of cases and 37(14.51) of controls were HIV positive. Among women who participated in the study, 6 (7.1%) of cases and 25 (9.8%) of controls had a history of sharing underwear with family, colleagues, and relatives. Concerning vaginal cleaning, 79 (92.9%) of cases and 243 (95.3%) controls have washed their vagina per day (Table 4).

**Table 4:** Sexual and behavioural characteristics of study participants at Debre Markos public health facilities, Northwest Ethiopia, 2025 (*n* = 340)

Variables	Category	Cases, *N* (%)	Controls, *N* (%)
Age at first sexual intercourse	< 18 years old	73 (85.8)	139 (54.5)
	*≥* 18 years old	12 (14.1)	116 (45.5)
History of STI	Yes	69 (81.1)	101 (39.70
	No	16 (18.8)	154 (60.4)
HIV status	Positive	40 (47.0)	37 (14.5)
	Negative	45 (52.9)	218 (85.5)
Lifetime sexual	One	13 (15.3)	155 (60.2)
	Two or more	72 (84.7)	100 (39.2)

*HIV *Human Immunodeficiency Virus, *STI *Sexually Transmitted Infections

### Factors associated with precancerous cervical lesions

In the Bivariable analysis, maternal age, marital status, residence, contraceptive use, menstrual history, genital mutilation, age at first sexual intercourse, sexually transmitted infection, HIV result status, and lifetime sexual partner were significant at a p-value of < 0.25. To control the effect of other confounding factors, they were entered into a multivariable logistic analysis. In the multivariable analysis, women aged > 40 years, rural residence, live alone marital status, age at first sexual intercourse less than 18 years old, history of female genital mutilation, history of STI, lifetime multiple sexual partners, and HIV status were found to be significant predictors of PCL at p-value < 0.05. Accordingly, women aged above 40 years had approximately three times higher odds of having PCL, AOR = 2.99 (95% CI: 1.03–8.90), compared to women aged less than 30 years. Women who had lived alone (single, divorced & widowed) were 4.86 times more likely to have PCL compared with those who were married, AOR = 4.86 (95% CI: 2.05–11.5). Similarly, women who had resided in rural areas were 3.69 times more likely to develop PCL lesions compared to their counterparts, AOR = 3.69 (95% CI: 1.46–9.36). Women with a history of STIs had 5.3 times higher odds of developing PCL compared to those without such a history, AOR = 5.3 (95%CI: 2.12–13.3). The odds of PCL were approximately six times higher among women who had genital mutilation compared with their counterparts, AOR = 5.98 (95% CI: 2.37–15.08). The odds of developing PCL were higher among women who initiated sexual intercourse before their 18th year compared to those who initiated first sexual intercourse at 18 and above years, AOR = 3.47 (95% CI: 1.27–9.5).

Similarly, women who had two or more lifetime sexual partners were 6.87 times more likely to develop precancerous cervical cancer compared to women who had only one lifetime sexual partner, AOR = 6.87 (95% CI: 2.74–17.2). Lastly, women who had HIV positive were 7.04 times more likely to develop precancerous cervical cancer than HIV-negative women, AOR = 7.04 (95% CI: 2.75–17.9) (Table 5).

**Table 5:** Logistic regression of precancerous cervical lesion and associated factors at Debre Markos town health facility, Northwest Ethiopia, 2025 (*n* = 340)

Variable	Category	Case *n*(%)	Control (%)	COR with 95%CI	AOR with 95% CI
Age in years	≤ 30	15(17.65)	75(29.41)	1	1
	31–40	21(24.71)	64(25.10)	1.64(0.78-34)	3.34(0.96-11.5)
	> 40	49(57.65)	116(45.49)	2.11(1.10–4.03)	2.99(1.03–8.90)*
Marital status	Live alone	67(78.82)	86(33.73)	7.31(4.08–13.08)	4.86(2.05–11.5)**
	Married	18(21.18)	169(66.27)	1	1
Residence	Rural	64(75.29)	96(37.65)	5.04(2.90–8.78)	3.69 (1.46–9.36) *
	Urban	21(24.71)	159(62.35)	1	1
Use Contraceptive	Yes	55(64.71)	87(34.12)	3.54(2.11–5.92)	2.25 (0.947 − 5.36)
	No	30(35.29)	168(65.88)	1	1
Menstrual history	Sometimes irregular	28(32.94)	64(25.10)	2.92(1.47–5.82)	2.47 (0.742 − 8.2)
	Always irregular	30(35.29)	69(27.06)	2.907(1.4–5.72)	2.52(0.845 − 7.55)
	No menses	11(12.94)	15(5.88)	4.90(1.91–12.5)	1.98(0.426 − 9.27)
	Regular	16(18.82)	107(41.96)	1	1
Genital mutilation	Yes	73(85.88)	86(33.73)	11.95(6.1–23.2)	5.98(2.37–15.08)**
	No	12(14.12)	169(66.27)	1	1
Age at first sexual intercourse	< 18 years	73(85.88)	139(54.51)	5.076(2.62–9.8)	3.47 (1.27–9.5) *
	*≥* 18 years	12 (14.12)	116(45.49)	1	1
History of STI	Yes	69(81.18)	101(39.69)	6.57(3.61–11.9)	5.3(2.12–13.3) **
	No	16(18.82)	154(60.39)	1	1
HIV result status	Positive	40(47.06)	37(14.51)	5.23(3.0- 9.08)	7.04 (2.75–17.9) **
	Negative	45(52.94)	218(85.49)	1	1
Lifetime sexual partner	Only one	13(15.29)	155(60.22)	1	1
	Two or more	72(84.71)	100(39.22)	8.58(4.51–16.3)	6.87(2.74–17.2) **

1 indiated Reference

* indicated *P* < 0.05, and ** indicated *P* < 0.01

## Discussion

This study aimed to assess the determinants of precancerous cervical lesions among screened women at Debre Markos town public health facility. Hence, women who live alone, initiated sexual activity before the age of 18, have genital mutilation, have two or more Lifetime sexual partners, have a history of STI, and have HIV were found to be significant determinants of precancerous cervical lesions.

Accordingly, the higher odds of PCL were found among women who were 40 years old or above compared to those who were less than 30 years old. This association is supported by previous studies conducted in Ethiopia at East Gojjam [35]., Addis Ababa [15], Woliso [18], and Mekelle [36], . This may be because the immune system’s ability to eradicate HPV infections can also decrease with age. Advanced maternal age reduces infection resistance and contributes to the development of disease [37]. In contrast, in Rwanda [38] and Cameroon [39], the lower odds of PCL were found among women above 40 years old [38]. This might be due to the methodological differences, which may be attributed to variations in screening programs, cultural practices, or sample sizes.

Marital status was identified as a determinant of PCL. Women who had lived alone (single, divorced, widowed) were nearly five times more likely to have the PCL compared to those who were married. This association is supported by several previous studies conducted in Addis Ababa, Mekelle, and Rwanda, which similarly reported a significant association between unmarried status and increased risk of PCL [14, 36, 38]. This might be because unmarried women may have higher exposure to multiple sexual partners, which increases the risk of acquiring STIs, including HPV, the principal etiological factor for cervical carcinogenesis. In addition to this, women living alone may encounter social, economic, or healthcare-related barriers that reduce their utilization of CC screening and follow-up services. Limited access to early screening and timely treatment may facilitate the persistence and progression of HPV to PCL [40]. Furthermore, reduced partner support and lower health-seeking behavior among unmarried women may also contribute to the delayed detection and management of PCL [41].

The higher odds of PCL were found among women who resided in rural areas compared to their urban counterparts. This association is supported by previous studies conducted in the East Gojjam zone, in Ethiopia [35]. This might be because rural women were more affected by risk behaviours or conditions such as early marriage, early sexual debut, and genital mutilation, all of which may increase susceptibility to persistent human papillomavirus HPV infection and subsequent cervical epithelial abnormalities. Furthermore, rural women often face substantial barriers in accessing cervical cancer prevention services, poor transportation infrastructure, and lower healthcare utilization [42].

Having STIs and HIV/AIDS was positively associated with the occurrence of PCL. Accordingly, the higher odds of PCL were found among women who had STIs and HIV compared to their counterparts. This association was supported by previous studies conducted in Bahir Dar Town [31], north Shoa in Amhara [17], Woliso town [18], Oromia region [16], a study conducted in Ethiopia [8], and Swaziland [43]. This might be due to chronic inflammation caused by STDs, which raises the likelihood of cervical precancerous lesions. This could be related to the fact that women with a history of STIs have a higher risk of being infected with HPV. The human papillomavirus is the most well-known risk factor for cervical cancer [44, 45]. Furthermore, HIV-positive women show a markedly increased risk, consistent with the immunosuppressed state facilitating HPV persistence and lesion development. The immunocompromised state facilitates persistent HPV infection, accelerates cervical cellular abnormalities, and increases the likelihood of progression to PCL [44]. The wide CI in HIV may indicate imprecision due to the small number of HIV positive controls (*n* = 37).

Besides, the current study showed that women who had more than one lifetime sexual partner been at a higher risk of developing PCL than women who had only one lifetime sexual partner. This finding was comparable with a study conducted in Addis Ababa, Woliso town, Bahir Dar, and Swaziland [8, 15, 18, 31, 43] that multiple sexual partners were a determining factor for the development of PCL. The increased likelihood of exposure to high-risk HPV infection among women with multiple sexual partners may explain the observed association. Women with multiple sexual partners may have a greater probability of exposing HPV infection through repeated sexual exposure, thereby increasing persistent infection and subsequent cervical epithelial changes. Furthermore, having multiple sexual partners may indirectly reflect engagement in high-risk sexual behaviours, including inconsistent condom use, early sexual activity, and having sex with casual partners, all of which increase the risk of PCL [46].

In the current study, women who initiated first sexual intercourse before the age of 18 were at a higher risk of the development of PCL. The findings of this study were consistent with studies in North Shoa, Amhara [17], Woliso Town [18], Arba Minch [47], Adama [13], and Rwanda [38], in which women who had a history of early initiation of first sexual intercourse before 18 years and less were at higher risk of developing PCL. Puberty might be the cause of this, as the ectocervix’s transformation zone enlarges, cervical tissue changes physiologically, and exposure to HPV can lead to infection and the development of dysplasia, a cervical squamous precancerous condition. This is the reason why HPV infection typically reaches its peak transmission early in the first year following the initiation of sexual activity [48]. Moreover, early initiation of sexual intercourse is at risk of contracting STIs, including HPV, related to a weak immune system and fragile vaginal mucosa [49].

Lastly, in the current study, FGM was significantly associated with PCL. Hence, women who had genital mutilation were 5.98 times more likely to have PCL than those who had no genital mutilation. This study was supported by the study conducted in Sudan [21], and Senegal [22]. This is because any trauma to the female genital organs is a predisposing factor to infection. Female genital organ trauma, such as episiotomy, cervical laceration, and genital mutilation, increases the risk of infection with HPV, a fundamental risk factor for CC [50]. Therefore, healthcare workers and program planners shall focus on strengthening targeted CC screening for high-risk groups, integrating screening services with HIV and STI clinics, improving reproductive health education, increasing awareness regarding harmful traditional practices such as FGM, and expanding screening access for rural women.

### Policy and practical implications of the study

This study provides essential evidence for healthcare workers, program planners, and policymakers working on CC prevention and reproductive health in Ethiopia. The identification of modifiable key determinants such as STIs, HIV, multiple sexual partners, early initiation of sex, and FGM highlights the need for targeted and integrated CC prevention strategies. Expanding the integration of CC screening services with STIs and HIV clinics could improve early detection and management of CC. Furthermore, the findings emphasize the importance of strengthening HPV vaccination, sexual and reproductive health education, and community awareness, focusing on the prevention of FGM. From a practical point of view, healthcare workers should prioritize regular screening and close follow-up, particularly among women with HIV, a history of STIs, and those with multiple sexual partners. These interventions could substantially reduce the burden of PCL- and CC-related morbidity and mortality in Ethiopia.

### Strengths of the study

This study has several notable strengths. The use of an analytical case-control study design enabled the identification of factors associated with the outcome of interest. Inclusion of multiple public health facilities enhanced the representativeness and generalizability of the findings within the study setting. Moreover, the study employed an adequate sample size and a high response rate, which strengthened the statistical power and reliability of the results. Importantly, the study assessed underexplored socio-cultural determinants, such as FGM, in the Ethiopian context, thereby providing valuable evidence to the existing body of literature.

### Limitations of the study

Although this study highlights important determinants of PCL, it has some limitations. Consecutive sampling of cases (rather than random) may introduce selection bias. In addition, self-reports of female genital mutilation, STIs history, and sexual behaviour were subjected to recall and social desirability bias. Moreover, the study was conducted in health facilities of a single town, which may limit the generalizability of the findings. Lastly, a relatively wide CI in some variables, which may indicate limited precision due to smaller subgroup sizes among categories.

## Conclusion

The study highlights the multifactorial nature of the PCL among women in Ethiopia, indicating the combined influence of biological, behavioural, and socio-cultural determinants. Women with a history of STIs, multiple sexual partners, early sexual initiation, FGM, and living with HIV were identified as particularly vulnerable groups requiring priority attention in CC prevention. These findings highlight the need for targeted and integrated interventions that enhance CC screening within HIV and STIs, particularly for high-risk and rural populations. Moreover, improving awareness of reproductive health risks and culturally sensitive community-based interventions aimed at reducing FGM are essential to strengthen early detection and prevention efforts. Future longitudinal and HPV-related molecular studies are recommended to better understand the causal pathways and further explore the socio-cultural determinants associated with PCL.

## Data Availability

The relevant data in this study are available in the manuscript and from the corresponding author upon request.

## Abbreviations

AOR: Adjusted Odds Ratio
BSC: Bachelor's Degree of Science
CC: Cervical Cancer
DNA: Deoxyribonucleic Acid
FGM: Female Genital Mutilation
FMOH: Federal Ministry of Health
HIV: Human immunodeficiency Virus
HPV: Human Papilloma Virus
IUCD: Intra Uterine Contraceptive Device
PAP SMEAR: Papanicolaou SMEAR
PCL: Pre-cancerous Cervical Lesions
SCJ: Squamocolumnar Junction
STI: Sexually Transmitted Infections
SSA: Sub-Saharan Africa
VIA: Visual Inspection with Acetic acid
